# Vasopressin Surrogate Marker Copeptin as a Potential Novel Endocrine Biomarker for Antidepressant Treatment Response in Major Depression: A Pilot Study

**DOI:** 10.3389/fpsyt.2020.00453

**Published:** 2020-05-20

**Authors:** Agorastos Agorastos, Anne Sommer, Alexandra Heinig, Klaus Wiedemann, Cüneyt Demiralay

**Affiliations:** ^1^ Department of Psychiatry and Psychotherapy, University Medical Center Hamburg-Eppendorf, Hamburg, Germany; ^2^ Department of Psychiatry, Division of Neurosciences, Faculty of Health Sciences, School of Medicine, Aristotle University of Thessaloniki, Thessaloniki, Greece; ^3^ VA Center of Excellence for Stress and Mental Health (CESAMH), VA San Diego Healthcare System, San Diego, CA, United States

**Keywords:** copeptin, vasopressin (AVP), depression, hypothalamus-pituitary-adrenal axis (HPA axis), cortisol, biomarker, treatment response, antidepressants

## Abstract

**Background:**

Major depressive disorder (MDD) constitutes the leading cause of disability worldwide. Although efficacious antidepressant pharmacotherapies exist for MDD, only about 40–60% of the patients respond to initial treatment. However, there is still a lack of robustly established and applicable biomarkers for antidepressant response in everyday clinical practice.

**Objective:**

This study targets the assessment of the vasopressin (AVP) surrogate marker Copeptin (CoP), as a potential peripheral hypothalamic-level biomarker of antidepressant treatment response in MDD.

**Methods:**

We measured baseline and dynamic levels of plasma CoP along with plasma ACTH and cortisol (CORT) in drug-naive outpatients with MDD before and after overnight manipulation of the hypothalamic-pituitary-adrenal (HPA) axis [i.e., stimulation (metyrapone) and suppression (dexamethasone)] on three consecutive days and their association with treatment response to 4 weeks of escitalopram treatment.

**Results:**

Our findings suggest significantly higher baseline and post-metyrapone plasma CoP levels in future non-responders, a statistically significant invert association between baseline CoP levels and probability of treatment response and a potential baseline plasma CoP cut-off level of above 2.9 pmol/L for future non-response screening. Baseline and dynamic plasma ACTH and CORT levels showed no association with treatment response.

**Conclusions:**

This pilot study provide first evidence in humans that CoP may represent a novel, clinically easily applicable, endocrine biomarker of antidepressant response, based on a single-measurement, cut-off level. These findings, underline the role of the vasopressinergic system in the pathophysiology of MDD and may represent a significant new tool in the clinical and biological phenotyping of MDD enhancing individual-tailored therapies.

## Introduction

Major depressive disorder (MDD) is a debilitating disease of high lifetime prevalence and constitutes worldwide the leading cause of disability due to chronic disease burden as it is significantly associated with a broad range of physical co-morbidities and higher overall mortality (1–3). Despite some controversial debates (4), pharmacotherapy with antidepressant agents is considered one of the best-established, efficacious, and widely used first-line treatments for MDD (5). Nevertheless, only about 40–60% of the patients respond to initial antidepressant therapy, while only approximately a third will reach remission and about 30–40% may experience treatment resistance even when receiving optimal antidepressant treatment according to consensus guidelines (6–8). Medication switch and augmentation strategies are then often employed, however with decreasing effectiveness at each successive treatment step (7, 9). As poor response is associated with higher functional impairment, mortality, morbidity, and chronicity of the disorder in the long term (10, 11), the identification of robust and generally applicable predictive factors of antidepressant non-response is highly warranted. However, besides some general negative clinical and psychosocial predictors of non-response (12), there is a lack of objective, individualized, and applicable biomarkers able to distinguish pathophysiological subgroups of depressed patients with different response to antidepressant treatment in everyday clinical practice (13).

Pathophysiologically, MDD is considered a stress-related disorder with distinct neuroendocrine profile (14), in particular showing alterations in the physiological function of the hypothalamic-pituitary-adrenal (HPA) neuroendocrine axis (2, 15). Hereby, HPA axis hyperactivity with higher cortisol (CORT) levels, increased corticotropine releasing hormone (CRH), and adrenocortocotropic hormone (ACTH) activity, attenuated CORT awakening response and altered feedback-circuits with reduced glucocorticoid receptor sensitivity are among the most consistent and robust findings (2, 15–20). In addition, antidepressant treatment leading to remission of the depressive psychopathology has been shown to be associated to normalization of the altered neuroendocrine stress system regulation (20–22). HPA axis functioning has been, accordingly, extensively studied using neuroendocrine challenge tests that attenuate or enhance CORT, ACTH, and CRH release (i.e., HPA axis suppression or stimulation) in terms of their potential predictive validity with respect to antidepressant response, as a subgroup of MDD patients with HPA axis alterations may be less likely to respond to treatment with antidepressants. Unfortunately, to date, and despite initial enthusiasm of pioneer studies (23), most results could not be adequately replicated and no pre-treatment basal or dynamic peripheral endocrine measure [i.e., baseline CORT, ACTH, CRH, assessment of the vasopressin (AVP), CORT/dehydroepiandrosterone ratio, dexamethasone (DEX) suppression test (DST), DEX/CRH test] was able to be robustly established as a valid and clinically applicable biomarker for antidepressant response (24–28).

However, the valid assessment of the hypothalamic level of the HPA axis functioning is challenging. The co-secreted and synergistically at this level acting CRH and AVP cannot be simply measured because of their pulsatile secretion pattern, small molecular size, avid binding to platelets, rapid plasma clearance, degradation, and instability (28, 29). In contrast, a surrogate marker of AVP secretion, the 39-amino-acid glycopeptide copeptin (CoP), is a more stable molecule in the circulation even at room temperature (*ex vivo*) and can be easily and reliably measured with a sandwich immunoassay (30, 31). CoP is the C-terminal cleavage part of the precursor pre-proAVP and is co-released in an equimolar ratio to AVP, thus closely mirroring AVP secretion, without a specific physiological function of its own (i.e., analogous to C-peptide for insulin) (32, 33). CoP has therefore emerged as a promising biomarker in several AVP-associated endocrine, cardiovascular, pulmonary, and renal disorders, as well as other acute medical stress states (e.g., sepsis) (30, 34–38).

However, to date, CoP has with few exceptions (39) not been regularly investigated in psychiatric research and, to date, no study has particularly assessed CoP as a treatment response biomarker in MDD. Thus, the main objective of our study was to assess CoP as a potential hypothalamic biomarker of antidepressant treatment response in MDD patients in order to investigate its predictive properties distinguishing responders from non-responders. Based on previous studies on HPA axis (re)activity (24, 26), we hypothesized that hyperactivity of the axis (i.e., vasopressinergic hyperdrive) with higher CoP plasma levels would be associated with poorer response to antidepressant treatment (40).

## Materials and Methods

### Study Participants and Inclusion Criteria

The study was approved by the ethics review committee (ERC) of the Hamburg Medical Board (file Nr. PV4161). Patients were recruited through our specialized depression outpatient clinic at the Department of Psychiatry and Psychotherapy, University Medical Center Hamburg-Eppendorf. After full oral and written explanation of the purpose and procedures of the investigation, written informed consent was obtained from each patient before initiating the screening procedure and enrollment in the study. Screening included a thorough physical and neurological examination, routine blood laboratory tests, urine toxicology screen, electrocardiogram (ECG), and a structured face-to-face clinical interview. Inclusion criteria included age of 18–65 years, a psychiatrist-confirmed diagnosis of non-psychotic MDD, single or recurrent, according to Diagnostic and Statistical Manual of Mental Disorders IV - Text Revision (DSM-IV-TR) criteria and an additional confirmatory minimum cut-off score for at least moderate depression on both the clinician- and self-rated Inventory of Depressive Symptomatology (IDS) questionnaire, as well as at least 8 weeks free of any psychiatric medication. Exclusion criteria included presence or self-reported history of any chronic or acute physical and axis I mental co-morbidities, history of psychotic MDD, body mass index (BMI) values beyond 18–30 kg/m^2^, frequent usage of any either illicit or prescribed drugs or over the counter medications, drinking of more than 100 g of alcohol per week, current adverse life events (e.g., divorce, loss of job, and illness in the family), night shifts or transcontinental flights across more than four time zones during the past 4 weeks, abnormal physical and neurological examinations, basic blood laboratory test values deviating from the normal range, positive urine toxicology screen, actual menstruation, pregnancy, nursing, or not using a reliable method of birth control, any contraindication for DEX or metyrapone, and pathological initial ECG. Hypothyroidism in the euthyroid state through hormonal substitution, as well as hypertension in normotensive state through antihypertensive medication, did not serve as exclusion criteria. Smoking status (smoking 1 cigarette/cigar/pipe or chewing tobacco 1x/day or more) has been assessed by self-report as a dichotomous variable (yes/no). We enrolled antidepressant-free patients with a clinical, non-psychotic MDD and without other physical and psychiatric comorbidities.

### Study Procedures

Patients who met study inclusion and exclusion criteria were scheduled for study initiation within 1 week of final laboratory results. The study assessed endocrine plasma levels of CORT, ACTH, and CoP at the morning of three consecutive days (same week-days for all patients: Monday–Wednesday) to measure HPA-axis activity after two endocrine challenges described below (challenge 1: Overnight Metyrapone Stimulation Test, MST; challenge 2: Overnight Low-Dose DST): day 1 (baseline), day 2 (post-MET), and day 3 (post-DEX). All subjects were encouraged to maintain a regular sleep time starting at around 11.00 p.m. for all three nights before study days 1–3, with wake-up at 7.00 a.m. and avoidance of physical strain (e.g. physical exercise, sexual activity, etc.) on all three study mornings. Furthermore, participants were encouraged to use public transportation or private motor vehicles [i.e. no bicycles or walking > 500 m to exclude to potential effects of elevated physical activity on heart rate (HR) measures] to reach the study facility and to avoid any intake of food or beverages (water was allowed) until completion of the assessment. After reaching the study facility (at 8.30 a.m.), all patients were given 15 min in sitting and 15 min in supine position in a single bedded room before the blood draw (09.00 a.m. of each study day).

Immediately after day 3 assessment, antidepressant pharmacotherapy with escitalopram (ESC) was initiated in all patients. ESC was selected as antidepressant agent of choice according to several international guidelines recommending selective serotonin reuptake inhibitors (SSRIs) as first-line treatment for depression (41–43) and some guidelines and meta-analyses noting possible superiority of escitalopram over other SSRIs with respect to efficacy, safety, tolerability, and cost (5, 41–43). Antidepressant treatment with ESC was initiated with an initial titration dosage scheme of 5 mg/d for 5 d and subsequent elevation and maintenance antidepressant treatment of 10–20 mg/d each morning. Treatment response was evaluated after a total of 4 weeks of maintenance treatment (i.e., total 32 d from starting date) through detailed clinical assessment including questionnaire evaluation as described below. Response to antidepressant treatment was defined as a reduction of 50% or more on both the initial IDS_30_-C and IDS_30_-SR depression scores.

### Endocrine Challenges

*- Overnight Metyrapone Stimulation Test (MST):* The MST test is considered to be a simple and sensitive alternative test to evaluate the ACTH reserve and is useful to evaluate the response of the HPA axis (44, 45). MET crosses the blood-brain barrier and reduces, not only at the adrenal glands but also within the brain, CORT levels by blocking the enzymatic conversion of 11-deoxy-CORT to CORT by CYP11B1 (11-beta-hydroxylase, P-450c11), the last step in the synthesis of CORT. This leads to a rapid fall of CORT and an accumulation of 11-deoxy-CORT, which does not inhibit ACTH secretion. This results in decreased CORT-mediated negative feedback at hypothalamic and pituitary levels, which increases CRH and ACTH secretion. Subjects received 1 g of MET (Metopiron^®^, Novartis, Arnhem, Netherlands) orally at 11.00 p.m. on day 1, to assess its effects on endocrine measures the next morning (day 2, approx. 9 h after metyrapone intake).

*- Overnight Low-Dose Dexamethasone Suppression Test (DST):* The low-dose DST is one of the commonly used tests to assess HPA axis reactivity by measuring the change in peripheral CORT levels in response to externally administered DEX (46). DEX is an exogenous steroid that binds mainly to glucocorticoid receptors in the anterior pituitary gland. This results in regulatory modulation through negative feedback and suppression of ACTH and consequently lowers CORT secretion (47). Subjects received 1 mg of DEX (Fortecortin^®^, Merck, Darmstadt, Germany) orally at 11.00 p.m. on day 2, to assess its effects on endocrine measures the next morning (day 3, approx. 9 h after DEX intake).

The temporal order of the two endocrine challenges was chosen to avoid any interference between the two interventions, as half-life times significantly differ between metyrapone (approx. 2 h) and DEX (35–54 h). Subjects were blinded with respect to the specific order of endocrine challenges.

### Laboratory Assays

Blood samples were obtained in EDTA coated tubes at 09.00 a.m. of each study day. Blood was placed in ice, plasma was separated and stored at −80°C until analysis. We determined CORT (CORT, ng/ml), adrenocorticotropic hormone (ACTH, pg/ml), and CoP (pmol/l) plasma levels using commercially available immunoradiometric assays and radio-immunoassays (DRG International Inc., USA; MP Biomedicals, Solon, USA; BRAHMS Kryptor, Berlin, Germany; respectively). The lower detection limit for CORT was 0.9 ng/ml and for ACTH was 5.7 pg/ml. Intra- and inter-assay coefficients of variation (CVs) were below 8% for all assays.

### Measures

All subjects underwent a detailed clinical assessment. Diagnosis of MDD, as well as exclusion of other current or lifetime comorbid axis I psychiatric disorders were established with the Structured Clinical Interview for the DSM-IV-TR Axis I Disorders (SCID-I) by experienced and specially-trained, board-certified psychiatrists. All other medical exclusion criteria were assessed in a clinical interview setting through study questionnaires. Depression severity was assessed using the German version of the IDS (48), a 30-item questionnaire with a clinical-rated (IDS_30_-C) and self-rated version (IDS_30_-SR), as it is proven to be more sensitive to changes in depressive psychopathology than other questionnaires (e.g., the Hamilton Depression Rating Scale) (49). The IDS is an assessment tool of excellent psychometric properties (total score range: 0–84) (50) that can be used to screen or assess the severity of depression and is widely used in large national and international multicenter studies and clinical trials both in- and outpatients (51), providing detailed information on depressive symptoms through 30 equally-weighted-items, including all nine Diagnostic and Statistical Manual (DSM) domains. Both an IDS_30_-C initial score of 23/24 and IDS_30_-SR initial score of 25/26 were used as cut-off scores, as indicated in prior literature (52), indicating clinical depression of at least moderate level and served as additional and confirmatory inclusion criterion to the study. Response to antidepressant treatment was defined as a reduction of 50% or more on both the initial IDS_30_-C and IDS_30_-SR depression scores. History of childhood maltreatment was assessed with the Childhood Trauma Questionnaire (CTQ) (53) and is presented as the total CTQ score (range: 25–125). Sleep quality was measured using the Pittsburgh Sleep Quality Index (PSQI) (54) and is presented as the total PSQI score. Adverse side effects were assessed through a German version of the UKU side effects rating scale (55).

### Statistical Analyses

Because several endocrine parameters showed skewed distribution, all endocrine parameters were ln-transformed for further parametric analysis. Preliminary analyses were performed to ensure no violation of the assumptions of normality, linearity and homoscedasticity. Ln-transformed parameters are presented as geometric means on the original scale through back-transformation through exponentiation of ln-transformed data. An error probability of *p* < 0.05 was accepted as statistically significant. Effect size is reported as partial eta squared (*_p_η^2^* = 0.01: small effect size, *_p_η^2^* = 0.06: medium effect size, *_p_η^2^* = 0.14: large effect size). To correct for potentially inflated type I error due to multiple comparisons we used the false discovery rate (FDR) approach (56), as in our previous studies (57–61). Following a previously reported procedure (62) *p*-values were corrected by the minimum positive FDR with a threshold set at 5%. Statistical analyses were conducted using the Statistical Package for Social Sciences Version 20 (SPSS, Chicago, IL).

## Results

One hundred seventy-five depressed patients were screened. Sixty-one patients were found eligible for participation in the study. Of those, 42 declined participation at the time of the scheduling phone call, 4 did not attend at scheduled appointment and 1 dropped out due to mild gastrointestinal side effects on day 1 by MET. We collected and analyzed data from 14 Caucasian study completers with MDD. Sample characteristics and psychometric scores can be found at **Table 1**. None of the subjects of the final sample was receiving additional medication (i.e. antihypertensive medication or thyroid hormone substitution). Side effects as per UKU ratings did not differ significantly between days indicating no significant adverse effects (data not shown). Of the 14 study completers, eight (57.1%) showed a treatment response after 4 weeks of ESC antidepressant treatment. The responder group did not differ from the non-responder group with respect to age, BMI, and initial depression, childhood trauma exposure, and sleep scores, nor did they differ in gender distribution, smoking status, and history of previous depression (**Table 1**). Mean ESC dosage was 17.1 (1.0; range: 10–20) mg/d in the total sample and did not differ significantly between the two groups [responders: 16.9 (1.3) mg/d; non-responders: 17.5 (1.7) mg/d; *t*_(12)_ = .295, *p* = .773)

**Table 1:** Demographic, physical, and psychometric measures in MDD patients and group differences between responders and non-responders to ESC antidepressant treatment.

Value	Total Sample	Responders	Non-Responders	*Responders vs. Non-Responders*		
	N (%)			*χ^2^*		*p*
**Participants**	14 (100.0%)	8 (57.1%)	6 (42.9%)			
**Males**	5 (35.7%)	2 (25.0%)	3 (50.0%)	.933		.580
**Smokers**	5 (35.7%)	3 (37.5%)	2 (33.3%)	.026		.999
**History of depression**	8 (57.1%)	5 (62.5%)	3 (50.0%)	.219		.957
	Mean (SEM)			*F*	*t*	*p*
**Age**	36.9 (2.3)	38.6 (2.3)	34.5 (4.5)	2.382	−.881	.396
**BMI**	26.1 (1.6)	24.5 (1.9)	28.2 (2.7)	.326	1.172	.264
**PSQI**	11.9 (1.1)	11.7 (1.4)	12.2 (2.0)	1.310	.190	.853
**CTQ**	41.4 (2.4)	41.5 (2.4)	41.2 (4.9)	.846	−.067	.948
**IDS-SR Baseline**	44.8 (2.0)	44.1 (2.7)	45.7 (3.2)	.492	.369	.719
**IDS-C Baseline**	42.8 (2.1)	42.4 (2.1)	43.2 (4.0)	.427	.171	.867
**IDS-SR Post-Treatment**	26.3 (3.0)	18.2 (2.6)	37.0 (1.7)	**1.024**	**5.598**	<.001***
**IDS-C Post-Treatment**	20.6 (2.9)	13.5 (2.5)	30.0 (3.0)	**.033**	**4.257**	.001**

Values are presented as total numbers (percent of total) and means (SEM). Psychometric scores report total scores. Age is reported in years. BMI, body mass index (kg/m^2^); PSQI, Pittsburgh Sleep Quality Index; CTQ, Childhood Trauma Questionnaire; IDS-SR, Inventory of Depressive Symptomatology–Self Report; IDS-C, IDS–Clinician. Group differences were assessed through t-test (parametric) or chi-squared test (nominal). Significant results are bolded. FDR analysis revealed no potential type I errors. **p < 0.01; ***p < 0.001.

**Figure 1:**
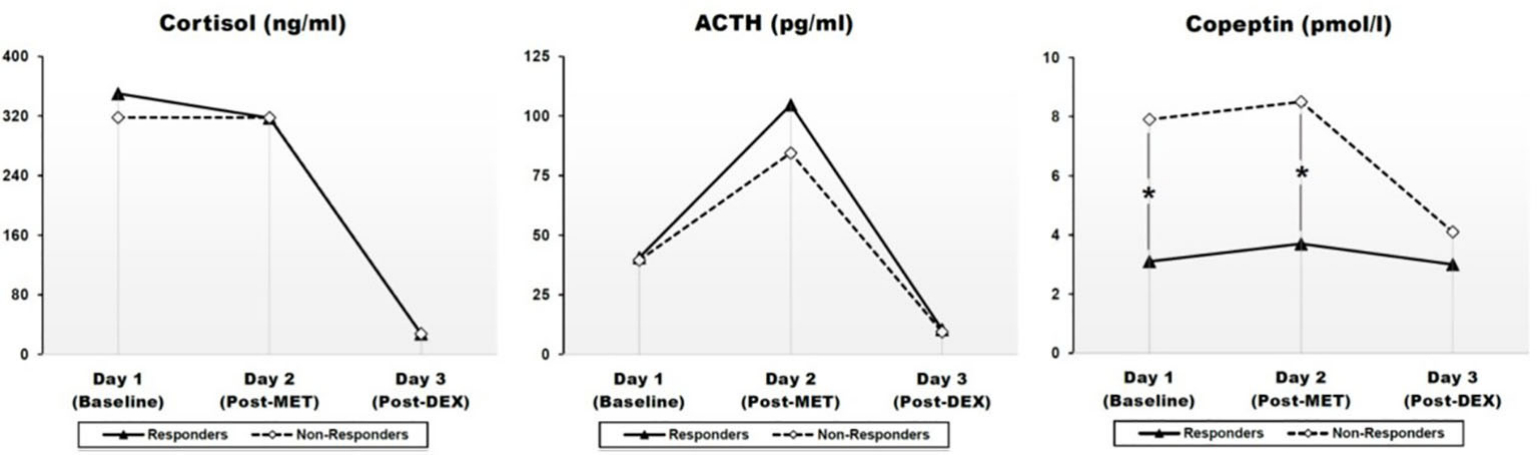
Effects of metyrapone (MET) and dexamethasone (DEX) on cortisol (CORT), ACTH, and CoP in responders and non-responders to ESC antidepressant treatment. Graphic presentation of **Table 2**. Pointwise values represent geometrical means adjusted for age, gender, BMI, history of previous depression and smoking. Group differences were assessed through a linear analysis of covariance (ANCOVA), controlling for age, gender, BMI, history of previous depression, and smoking. ACTH: Adrenocorticotropic hormone. Day 1: baseline; day 2: Post-MET: post-metyrapone; day 3: post-DEX: post-dexamethasone. A mixed between-within subject ANOVA indicated a highly significant effect of time (i.e. treatment condition) in all three measures with very large effect sizes, which confirmed the expected effects of each treatment condition (*cf*. *Results* section). Group (response vs. non-response) had a significant effect only for CoP, but not for CORT or ACTH, suggesting a statistically significant difference between responders and non-responders on CoP levels across days 1–3 (*cf*. *Results* section). *p < 0.05.

Adjusted geometrical means (SEM) of CORT, ACTH, and CoP levels across days 1–3 are presented at **Figure 1** and **Table 2**. There were no statistically significant differences with respect to CORT, ACTH, and CoP levels across days 1–3 with respect to gender, smoking status, medication dosage received, or history of previous depression (*data not shown*). There were also no statistically significant correlations between baseline CORT, ACTH, and CoP levels and psychometric scores, or their changes (*Δ*) through antidepressant treatment (*data not shown*).

**Table 2:** Adjusted geometric means of endocrine measures across the three conditions and group differences between responder and non-responder to escitalopram (ESC) antidepressant treatment.

Value	Day	Adjusted Geometrical Means (SEM)		*Controlled group differences* *(ANCOVA)*		
		Responders	Non-Responders	*F*	*p*	*_p_η^2^*
**CORT**	**Day 1: baseline**	350.0 (1.1)	318.0 (1.2)	.226	.651	.036
	**Day 2: post-MET**	317.3 (1.1)	317.7 (1.1)	.000	.998	.000
	**Day 3: post-DEX**	27.5 (1.1)	27.7 (1.2)	.001	.978	.000
**ACTH**	**Day 1: baseline**	40.6 (1.2)	39.5 (1.3)	.006	.940	.001
	**Day 2: post-MET**	104.7 (1.2)	84.5 (1.3)	.373	.564	.059
	**Day 3: post-DEX**	10.4 (1.1)	9.4 (1.1)	.527	.495	.081^+^
**CoP**	**Day 1: baseline**	3.1 (1.2)	7.9 (1.2)	**12.913**	.011*	**.683** ^++^
	**Day 2: post-MET**	3.7 (1.2)	8.5 (1.2)	**8.154**	.029*	**.576** ^++^
	**Day 3: post-DEX**	3.0 (1.3)	4.1 (1.3)	.778	.412	.115 ^+^

Values are presented as geometric mean values (SEM) adjusted for age, gender, history of previous depression, BMI and smoking. Group differences were assessed through a linear ANCOVA, controlling for age, gender, BMI, history of previous depression, and smoking. ACTH: Adrenocorticotropic hormone. Day 1: baseline; day 2: Post-MET: post-metyrapone; day 3: post-DEX: post-dexamethasone. Significant results are bolded. FDR analysis revealed no potential type I errors. ANCOVA, analysis of variance; CoP, copeptin; CORT, cortisol; DEX, dexamethasone; MET, metyrapone. ^+^ medium effect size, ^++^ = 0.14: large effect size. *p < 0.05.

A mixed between-within subject ANOVA was conducted to assess the difference between responders and non-responders on CORT, ACTH, and CoP levels across days 1–3. Results indicated a highly significant effect of time (i.e. treatment condition) in all three measures with very large effect sizes, which confirmed the expected effects of each treatment condition (CORT: Wilk's lamda = .015, F_(2,11)_ = 368.625, p < .001, *_p_η^2^ = .985; ACTH:* Wilk's lamda = .061, F_(2,11)_ = 85.189, p < .001, *_p_η^2^ = .939;* CoP: Wilk's lamda = .544, F_(2,11)_ = 4.616, p = .035, *_p_η^2^ = .456).* For CORT and ACTH, group (i.e., response vs. non-response) had no significant effect (CORT: F = .278, p = .608, *_p_η^2^ = .023; ACTH:* F = .271, p = .612, *_p_η^2^ = .022)*, nor was there any statistically significant group *x* time interaction (*data not shown*), suggesting no difference between responders and non-responders regarding CORT and ACTH levels across days 1–3. In contrast, for CoP, group had a significant effect on CoP levels across day 1–3 (F= 9.992, p = .008, *_p_η^2^ = .454*), although lacking significant group *x* time interaction (Wilk's lamda = .908, F_(2,11)_ = .560, p = .587, *_p_η^2^ = .092*), suggesting a statistically significant difference between responders and non-responders on CoP levels across days 1–3.

A logistic regression was performed to ascertain the effects of baseline plasma CoP levels on the likelihood of response to antidepressant treatment. The logistic regression model was statistically significant (*χ* ^2^_(1)_ = 7.784, *p* = .005). The model explained 57.3% (Nagelkerke *R2*) of the variance in treatment response and correctly classified 85.7% of cases. Higher baseline plasma CoP levels were significantly associated with a decreased likelihood of antidepressant treatment response (*B* = −.599, *SE* = .277, *Wald* = 4.226, *df* = 1, *p* = .040, *OR* = .572, 95% *CI* for *OR*:.336–.974). Every increase of baseline plasma CoP level of 1.0 pmol/L almost doubled the possibility of non-response. The relation between treatment response and plasma CoP levels was also examined using receiver operating characteristic (ROC) curves. The area under the curve was.823, which indicates that baseline plasma CoP levels are quite accurate in identifying response to antidepressant treatment. The ROC diagram suggested a plasma CoP cut-off level of 2.9 pmol/L (sensitivity 87.5%, specificity 83.3%) as the most appropriate one for screening purposes of non-response. A direct univariate logistic regression (*χ* ^2^_(1)_ = 5.004, *p* = .025, Nagelkerke *R2 = 40.3%)*, which correctly classified 78.6% of cases, indicated that depressed patients with a plasma CoP level above 2.9 pmol/L had a significantly higher probability of non-response (*B* = −2.708, *SE* = 1.366, *Wald* = 3.929, *df* =1, *Sig.* = .047, OR = .067, 95% *CI* for *OR*:.005–.970, FDR analysis indicated a potential type I error).

## Discussion

The main objective of our study was to assess the AVP surrogate marker CoP as a potential peripheral hypothalamic biomarker of treatment response in MDD patients and to investigate its predictive properties distinguishing responders from non-responders to standardized antidepressant treatment with ESC. We measured plasma CoP in MDD patients before and after manipulation of the HPA axis [i.e., stimulation (MET) and suppression (DEX)], along with plasma ACTH and CORT, in order to assess baseline and dynamic changes in all three levels of the axis and their correlation. To the best of our knowledge, this is the first study investigating plasma CoP as an endocrine predictor of antidepressant treatment in MDD. The main findings of the study include i) a significant difference between responders and non-responders between baseline and post-MET CoP levels, while no difference was apparent with respect to ACTH and CORT, ii) a statistically significant association between higher baseline plasma CoP levels and lower probability of treatment response, and iii) a baseline plasma CoP cut-off level above 2.9 pmol/L with a potential screening applicability in the differentiation between responder and non-responder.

Given that AVP is not considered a valid biomarker in MDD and psychiatric disorders in general (28), our results offer first evidence that plasma CoP may represent an alternative stable, easily accessible, and clinically applicable peripheral biomarker of antidepressant treatment response in MDD. CoP has been lately put forth as a possible novel stress marker as some studies have reported CoP to subtly mirror the individual stress level in psychological (63–65) and physical stress paradigms (66), while CoP has shown a positive correlation with other HPA axis hormones (64, 67–70). In a previous study of our research group (39), we could show that, following an objective, pharmacological panic challenge, plasma CoP not only delicately responded through a sharp increase to panic provocation and positively correlated with simultaneously assessed ACTH and CORT, but also significantly correlated with subjectively reported panic symptoms, which was not the case for ACTH and CORT. Our study found no correlation between CoP and ACTH and CORT, similarly to previous studies in MDD patients (67, 71). Taken together, this suggests that CoP can be reliably used as surrogate marker of AVP secretion and may even better represent the hypothalamic level of the HPA axis in stress-related disorders and MDD than CRH and AVP itself.

Our results are in accordance to the close association of the vasopressinergic system with HPA axis (re)activity and support a central role of the AVP neural system in the coordination of neuroendocrine responses to stress in the pathophysiology of MDD (40, 72–76). AVP modulates the release of ACTH and potentiates the effects of CRH on the pituitary, mainly through the G-protein-coupled vasopressin 1b (V_1b_) receptor and its expression (40, 74), while it is important to note that V_1b_ receptors have been additionally detected in the septum, cortex and hippocampus (77–79). Interestingly, AVP effects on HPA axis seem to differ between acute and chronic stress (80). Prolonged stress up-regulates AVP and pituitary V_1b_ expression (74, 81, 82), possibly through an impairment in the repression of the AVP promoter (83). Polymorphisms in the promoter structures of the AVP gene and V_1b_ receptor genes, could thus contribute to individual variation in stress resilience but also phenotypic expression of stress-related disorders and represent a promising target for pharmacotherapeutic interventions (75).

In MDD, an increased number of AVP-expressing neurons and increased AVP mRNA expression has been reported in the hypothalamic Periventricular Nucleus (PVN) (84, 85), along with elevated AVP plasma levels (86–90), confirming vasopressinergic hyperactivity with pituitary but also extra-hypothalamic and extra-pituitary AVP neuropeptidergic hyperdrive (40, 91). This AVP overdrive can lead to a “switch” in the regulation of the HPA system from CRH to AVP control, resulting in an altered homeostasis within the HPA system with different neurobiological, behavioral, and emotional effects (82) as seen in MDD (91, 92). Indeed, MDD is characterized by increased HPA responsiveness to AVP and decreased responsiveness to CRH (82), while antidepressant action is shown to be related to reduction of vasopressinergic overexpression in these patients (40, 93). In addition, higher AVP plasma levels in MDD have been associated with distinct depression features, such as melancholic symptoms and psychomotor retardation during the day (86, 90, 94). This is in accordance to our findings of higher CoP plasma levels in MDD patients showing future non-response in their clinical course, suggesting an association of a vasopressinergic hyperdrive with HPA axis-related, particularly burdening symptoms and negative outcome in MDD (76). It is, thus, not surprising that V_1b_ receptor antagonists, inhibiting AVP and CRH-induced ACTH release, have raised experimental interest as potential agents for the treatment of stress-related disorders and MDD (95). First clinical trials have supported anxiolytic- and antidepressant-like effects of such agents (e.g., SSR149415) in various animal-model and human clinical studies (96–100) and suggest that the V_1b_ receptor is required to drive the HPA axis response to acute antidepressant treatment (40, 101).

Finally, some limitations of our study merit discussion. The most important limitation is the number of patients included in the study. Because of rigid exclusion criteria, the combined endocrine challenge and its time-consuming nature, our study investigated only a relatively small number of MDD patients. Thus, our findings should be considered preliminary and should be replicated in larger study populations. Because of the limited patient sample, the design of the study included the use of only one specific antidepressant, in order not to introduce additional confounders. On the other hand, all subjects were extremely carefully selected to minimize the probability of medical (e.g., medication use, systemic and chronic diseases, inflammatory states, deviating laboratory, or physical tests) and behavioral (e.g., substance and alcohol use) confounders. We particularly accounted for several laboratory markers (e.g., fasting glucose, hemoglobin A1c levels, cholesterol/lipoproteins, pro-inflammatory cytokines, acute-phase proteins), subjective sleep problems, history of childhood trauma, BMI, gender, and other certain lifestyle habits (e.g., drug, alcohol or tobacco intake) that have been shown to be associated with alterations of the HPA axis (re)activity. Furthermore, our MDD patient sample reported similar depression scores as in reference studies (102), while their baseline morning CORT plasma levels were comparable to our prior studies on MDD patients (103–105), as was CoP (71), suggesting that we included a representative sample of MDD patients.

Secondary analyses investigating the impact of peri- and postmenopausal status (1 and 2 women, respectively) on our results, did not alter our findings (*data not shown*). However, although having excluded actual menstruation, we have not controlled for the specific menstrual phase of women participants. Additionally, it is very important to note that this study is measuring differences at defined time points of the day (i.e. single-time point measurements), which may relativize our findings. On the other hand, we assessed all individuals on three consecutive days, at the exact same time of day and the same week-days to minimize confounders. Finally, we acknowledge that nocturnal HPA axis stimulation/suppression leads to mainly indirect effects 9 h later. The complex pharmacokinetics of DEX and MET and their yet not fully understood interrelated pharmacodynamics, in addition to the nocturnal circadian phase with evolving different sleep stages and huge changes of HPA axis activity and circadian gene expression, represent important limitations with respect to the dynamic findings of this study.

## Conclusions

Better clinical and biological phenotyping of MDD through novel biomarkers could improve the identification of MDD subtypes with respect to antidepressant treatment response and help routine patient stratification for individual-tailored therapies, which could improve response rates and shorten disease duration (106). To date, no specific biomarker has managed to establish clinical relevance in routine clinical practice. Our findings provide first evidence in humans that the AVP surrogate marker CoP might represent a novel, easily accessible and clinically applicable biomarker of excellent predictive properties, which can correctly identify treatment response in MDD patients through a single measurement based on a cut-off level. These findings, if replicated, might help establish a significant new clinical tool in the management of MDD and underline the role of the vasopressinergic system in the pathophysiology of MDD and treatment non-response.

## Data Availability Statement

The datasets generated for this study are available on request to the corresponding author.

## Ethics Statement

The studies involving human participants were reviewed and approved by ethics review committee (ERC) of the Hamburg Medical Board (file Nr. PV4161). The patients/participants provided their written informed consent to participate in this study.

## Author Contributions

AA, CD, and KW designed the study and wrote the protocol. AA, AS, AH, and CD collected the data. AS and AA managed recruitment and screening of patients, as well as all literature searches. AA, AS, AH, and CD had access to the raw data. AA, AS, and AH performed all data processing and analyses. AA performed all statistical analyses and interpretation. AA and AS wrote the first draft of the paper. KW and CD revised the draft for important intellectual content. All authors have contributed to, read and approved the final version of the manuscript.

## Conflict of Interest

The authors declare that the research was conducted in the absence of any commercial or financial relationships that could be construed as a potential conflict of interest.
